# EBAG9/RCAS1 in human breast carcinoma: a possible factor in endocrine–immune interactions

**DOI:** 10.1054/bjoc.2001.2176

**Published:** 2001-11

**Authors:** T Suzuki, S Inoue, W Kawabata, J Akahira, T Moriya, F Tsuchiya, S Ogawa, M Muramatsu, H Sasano

**Affiliations:** Department of Pathology, Tohoku University School of Medicine, Sendai, Japan; Department of Biochemistry, Saitama Medical School, Saitama, Japan

**Keywords:** breast, carcinoma, EBAG9/RCAS1, immunohistochemistry, lymphocyte, oestrogen

## Abstract

EBAG9 has been recently identified as an oestrogen responsive gene in MCF-7 human breast carcinoma cells. EBAG9 is identical to RCAS1, a cancer cell surface antigen possibly involved in immune escape. In this study, we examined the expression of EBAG9/RCAS1 in human breast carcinomas using immunohistochemistry and reverse transcription-polymerase chain reaction (RT-PCR). EBAG9 immunoreactivity was also associated with various clinicopathological parameters, including intratumoural infiltration of inflammatory cells, to examine the biological significance of EBAG9 in human breast carcinomas. EBAG9 immunoreactivity was detected in the entire surface and cytoplasm of carcinoma cells in 82 out of 91 invasive ductal carcinomas (90.1%). In non-neoplastic mammary glands, EBAG9 immunoreactivity was weakly present on the luminal surface of epithelial cells. Results from RT-PCR (*n* = 7) were consistent with those of immunohistochemistry. EBAG9 immunoreactivity was significantly associated with estrogen receptor (ER) α labelling index (*P* = 0.0081), and inversely associated with the degree of intratumoural infiltration of mononuclear cells (*P* = 0.0020), or CD3^+^ T lymphocytes (*P* = 0.0025). This study suggests that EBAG9 is produced via ER in carcinoma cells and inhibits the intratumoural infiltration of T lymphocytes in the context of a possible endocrine–immune interaction in human breast carcinomas. © 2001 Cancer Research Campaign http://www.bjcancer.com


British Journal of Cancer (2001) 85(11), 1731–1737
© 2001 Cancer Research Campaign
doi: 10.1054/ bjoc.2001.2176, available online at http://www.idealibrary.com on IDEAL

### EBAG9/RCAS1 in human breast carcinoma: a possible factor in endocrine–immune interactions

**T Suzuki[1], S Inoue[2], W Kawabata[1], J Akahira[1], T Moriya[1], F Tsuchiya[2], S Ogawa[2], M Muramatsu[2] and H Sasano[1]**

[1]Department of Pathology, Tohoku University School of Medicine, Sendai, Japan; [2]Department of Biochemistry, Saitama Medical School, Saitama, Japan

**Summary** EBAG9 has been recently identified as an oestrogen responsive gene in MCF-7 human breast carcinoma cells. EBAG9 is identical to RCAS1, a cancer cell surface antigen possibly involved in immune escape. In this study, we examined the expression of EBAG9/RCAS1 in human breast carcinomas using immunohistochemistry and reverse transcription-polymerase chain reaction (RT-PCR). EBAG9 immunoreactivity was also associated with various clinicopathological parameters, including intratumoural infiltration of inflammatory cells, to examine the biological significance of EBAG9 in human breast carcinomas. EBAG9 immunoreactivity was detected in the entire surface and cytoplasm of carcinoma cells in 82 out of 91 invasive ductal carcinomas (90.1%). In non-neoplastic mammary glands, EBAG9 immunoreactivity was weakly present on the luminal surface of epithelial cells. Results from RT-PCR ($n$ = 7) were consistent with those of immunohistochemistry. EBAG9 immunoreactivity was significantly associated with estrogen receptor (ER) $\alpha$ labelling index ($P$ = 0.0081), and inversely associated with the degree of intratumoural infiltration of mononuclear cells ($P$ = 0.0020), or $CD3^+$ T lymphocytes ($P$ = 0.0025). This study suggests that EBAG9 is produced via ER in carcinoma cells and inhibits the intratumoural infiltration of T lymphocytes in the context of a possible endocrine–immune interaction in human breast carcinomas. © 2001 Cancer Research Campaign http://www.bjcancer.com

**Keywords**: breast; carcinoma; EBAG9/RCAS1; immunohistochemistry; lymphocyte; oestrogen

Breast carcinoma is one of the most common malignancies in women worldwide. Human breast tissue is a target for oestrogens, and these sex steroids have an important role to play in the development of hormone-dependent breast carcinomas (Thomas, 1984; Vihko and Apter, 1989). The biological effects of oestrogens are mediated through an initial interaction with the oestrogen receptor (ER), a member of the superfamily of nuclear receptors. Recently, a second ER, ER$\beta$, has been identified in humans (Kuiper et al, 1996; Mosselman et al, 1996), and the previously known human ER has been renamed ER$\alpha$ (Mosselman et al, 1996; Enmark et al, 1997). ERs are known to function as dimers, and to activate transcription in a ligand-dependent manner by binding to oestrogen-responsive elements (EREs) located in the promoter region of various target genes (Tsai and O'Malley, 1994). Therefore, it is very important to examine the expression of oestrogen-responsive genes to obtain a better understanding of oestrogenic actions and their regulation in hormone-dependent breast carcinomas.

Recently, EBAG9 (oestrogen receptor-binding fragment-associated gene 9) has been identified as an oestrogen-responsive gene from a cDNA library of MCF-7 human breast cancer cells (Watanabe et al, 1998). EBAG9 is identical to the receptor-binding cancer antigen expressed in SiSo cells (RCAS1) (Nakashima et al, 1999). RCAS1 is expressed in uterine, ovarian and lung cancers and has been suggested to be associated with a poor prognosis in these patients (Sonoda et al, 1996; Kaku et al, 1999; Iwasaki et al, 2000). EBAG9/RCAS1 is a membrane molecule that acts as a ligand for a putative receptor present in cells (Nakashima et al, 1999). In vitro studies have also demonstrated that EBAG9/RCAS1 inhibits growth of activated $CD3^+$ T lymphocytes, suggesting a possible involvement in the immune escape of neoplastic cells (Nakashima et al, 1999). Endocrine–immune interactions are considered to play an important role in the development and/or progression of various hormone-dependent human neoplasms, but the details of these interactions remain unclear. EBAG9 may play an important role in the development of oestrogen-dependent breast carcinomas, possibly through modulation of intratumoural immune environment. However, the expression of EBAG9 has not been examined in breast carcinoma tissues, and thus the biological significance remains unknown. Therefore, in this study, we examined the expression of EBAG9 in human breast carcinomas using immunohistochemistry and reverse transcription-polymerase chain reaction (RT-PCR). In addition, we statistically correlated the immunoreactivity of EBAG9 with various clinicopathological parameters in 91 cases of human breast carcinoma in order to study the possible roles of EBAG9 in hormone-dependent breast cancer.

*Received 30 May 2001*
*Revised 24 September 2001*
*Accepted 25 September 2001*

*Correspondence to:* T Suzuki

### MATERIALS AND METHODS

### Patients and tissues

91 specimens of invasive ductal carcinoma of the breast were obtained from female patients who underwent mastectomy from 1984 to 1989 in the Department of Surgery, Tohoku University Hospital, Sendai, Japan. Metastatic lesions in the resected lymph nodes were also examined in this study. The mean age of patients from whom specimens were obtained was 53.7 years (range 27–82). All patients examined in this study received neither irradiation nor chemotherapy prior to surgery. The clinical data, including patient age, menopausal status, stage according to UICC

TNM classification (1987), tumour size, and lymph node status, were retrieved from patient charts. The mean follow-up time was 104 months (range 15–154 months). Disease-free survival data were available for all patients. The histological grade of each specimen was evaluated by 3 of the authors (TS, TM, and HS), based on the modified method of Bloom and Richardson (1957), according to Elston and Ellis (1991). The intratumoural mononuclear infiltration grade of carcinomas was assessed as 0 (no areas), 1 (scattered small foci), 2 (scattered large foci), and 3 (numerous large or broad areas with pertinent changes), based on the method of Hamlin (1968) by these 3 authors.

All specimens were fixed with 10% formalin and embedded in paraffin-wax in the Department of Pathology, Tohoku University Hospital, Sendai, Japan.

### Antibodies

EBAG9 antibody was a rabbit polyclonal antibody against a GST-EBAG9 fusion protein (Tsuchiya et al, 2001). The Polyclonal antibody for ERβ was raised in rabbit against synthesized peptides of the C-terminal region of ERβ (CSPAEDSKSKEGSQNPQSQ) (Ogawa et al, 1998b). These antibodies were purified on affinity columns bound with the synthetic peptides. The characterization of these antibodies was confirmed by Western blotting (Ogawa et al, 1998b; Tsuchiya et al, 2001), and utilization of the ERβ antibody for immunohistochemistry has been previously reported (Takeyama et al, 2001). Monoclonal antibodies for ERα (ER1D5), PR (MAB429), and Ki-67 (MIB1) were purchased from Immunotech (Marseille, France), Chemicon (Temecula, CA, USA), and Immunotech (Marseille, France), respectively. Rabbit polyclonal antibodies for CD3 (A0452), which recognizes T lymphocytes, and HER-2/*neu* (A0485) were obtained from DAKO (Carpinteria, CA, USA).

### Western blot analysis

For characterization of EBAG9 antibody, Western blot analysis was performed using recombinant EBAG9 protein. Full lengths of mouse and human EBAG9 ORF cDNA were ligated into eukaryotic expression vector pcDNA3 (Invitrogen), and then mouse and human EBAG9 proteins were produced using TNT 17 Quick Coupled Transcription/Translation System (Promega, Madison, WI, USA). Western blot analysis was performed as described (Ogawa et al, 1998b). Briefly, 10 μg of each sample was fractionated on SDS-12.5% polyacrylamide gels, and electrophoretically transferred onto polyvinylidene difluoride (PVDF) membranes (Immobilon, Millipore Corp, Bedford, MA, USA). The membranes were blocked at room temperature for 1 h in phosphate-buffered saline (PBS) with 3% skim milk, then incubated at room temperature for 1 h with 5 ml each of 1:2000 diluted purified anti EBAG9 antibody, which was incubated with 0.1, 1, 10 and 100 μg of GST-EBAG9 fusion protein at 4˚C for 12 h. Each membrane was washed in PBS with 0.1% Tween 20 and incubated with 1:5000 diluted horseradish peroxidase-conjugated donkey anti-rabbit IgG (Amersham Pharmacia Biotech) at room temperature for 1 h. Bands were visualized with the chemiluminescence-based ECL™ or ECL™ plus detection system (Amersham Pharmacia Biotech). The membranes were exposed to X-ray film or placed on the STORM™ system (Molecular Dynamics, Sunnyvale, CA, USA). To detect the distribution of EBAG9 at the protein level, tissue of 8-week ICR mice purchased from SLC (Shizuoka, Japan) were collected and homogenized in lysis buffer (5 mM phosphate buffer, pH 7.4, with 0.1% Triton X-100), then 10 μg of each sample was used for Western blot analysis.

### Immunohistochemistry

Immunohistochemical analysis was performed by employing the streptavidin–biotin amplification method using a Histofine Kit (Nichirei, Tokyo, Japan) for EBAG9, ERα, PR, Ki-67, HER-2/*neu*, and CD3, or EnVision$^+$ (DAKO, Carpinteria, CA, USA) for ERβ in this study. For antigen retrieval, the slides were heated in an autoclave at 120˚C for 5 min in citric acid buffer (2 mM citric acid and 9 mM trisodium citrate dehydrate, pH 6.0) for the immunostaining of EBAG9, ERα, ERβ, PR, Ki-67, and HER-2/*neu* or incubated with 0.05% protease (Type XXVII; Sigma, St Louis, MO, USA) in 50 mM Tris-HCl buffer, pH 7.6 for 10 min for CD3, following deparaffinization. The dilutions of primary antibodies used in this study were as follows: EBAG9; 1/200, ERα; 1/2, ERβ; 1/100, PR; 1/30; Ki-67; 1/50, HER-2/*neu*; 1/200, and CD3; 1/500. The antigen–antibody complex was visualized with 3.3′-diaminobenzidine (DAB) solution (1 mM DAB, 50 mM Tris-HCl buffer (pH 7.6), and 0.006% $H_2O_2$), and counterstained with methyl green. For negative controls, normal rabbit or mouse IgG was used instead of the primary antibodies. For EBAG9 and ERβ, immunohistochemical preabsorption tests were also performed. No specific immunoreactivity was detected in these sections.

### RT-PCR

RT-PCR analysis was performed on 7 specimens of invasive ductal carcinoma. Total RNA was extracted by homogenizing tissue specimens in guanidinium thiocyanate followed by ultracentrifugation in cesium chloride, and quantified spectrophotometrically at 260 nm. A cDNA synthesis kit (SUPERSCRIPT Preamplification system, Gibco-BRL, Grand Island, NY, USA) was employed in the synthesis of first-strand cDNA. cDNAs were synthesized from 5 μg of total RNA using random hexamer primer. Reverse transcription was carried out for 60 min at 42˚C with SUPERSCRIPT II reverse transcriptase. After an initial 1-min denaturation step at 95˚C, 40 cycles of PCR were carried out on a LightCycler (Roche Diagnostics GmbH, Mannheim, Germany) under the following conditions: 1-min denaturation at 95˚C, 15-s annealing at 58˚C for EBAG9, 62˚C for ERα, or 60˚C for ERβ and β-actin, and 15-s extension at 72˚C. The primer sequences used in this study are as follows: EBAG9 (FWD 5′ and REV 5′-CTTCTTCATTAGCCGTTGTG), ERα (FWD 5′-AAGA GCTGCCAGGCCTGCC and REV 5′-TTGGCAGCTCT-CATGTCTCC) (Pujol et al, 1998), ERβ (FWD 5′-GCATGGAA-CATCTGCTCAAC and REV 5′-ACGCTTCAGCTTGTGAC-CTC) (Ogawa et al, 1998a), and β-actin (FWD 5′-CCAACCGC-GAGAAGATGAC and REV 5′-GGAAGGAAGGCTGGAA-GAGT). Oligonucleotide primers for EBAG9 and β-actin were designed using perviously published cDNA sequences for human EBAG9 (Watanabe et al, 1998) and β-actin (Ponte et al, 1984), respectively. Following PCR, the products were resolved on a 2% agarose eithidium bromide gel, and images were captured with Polaroid film under UV transillumination. In initial experiments, following amplification, PCR products were purified and subjected to direct sequencing (ABI PRISM BigDye Terminator Cycle Sequencing Ready Reaction Kit, Perkin Elmer Applied Biosystems, Foster City, CA, USA; and ABI Prism 310 Genetic Analyzer) to verify amplification of the correct sequences. As a

 *© 2001 Cancer Research Campaign*

positive control, MCF-7 breast cancer cells were used for EBAG9 (Watanabe et al, 1998) and ERα (Vladusic et al, 2000), and T-47D breast carcinoma cells were used for ERβ (Vladusic et al, 2000). Negative control experiments lacked the cDNA substrate to check for the possibility of exogenous contaminant DNA. No amplified products were observed under these conditions.

### Scoring of immunoreactivity

For statistical analyses of EBAG9 and HER-2/*neu* immunoreactivity, carcinomas were classified independently by 3 of the authors (TS, TM and HS) into 2 groups: +, positive carcinoma cells; and –, no immunoreactivity. Cases with disconcordant results among the observers were re-evaluated. Scoring of ERα, ERβ, PR and Ki-67 in carcinoma cells was performed on high-power fields ( × 400) using a standard light microscope. In each case, more than 500 carcinoma cells were counted independently by these same 3 authors, and the percentage of immunoreactivity, i.e. labelling index (LI), was determined. In the present study, inter-observer differences were less than 5%, and the mean of the 3 values was obtained. To determine the infiltration of T lymphocytes, 3 of the authors (TS, TM and HS) independently counted the number of $CD3^+$ cells on high-power fields ( × 400) using a standard light microscope. For each specimen, 6 fields were selected at random in the invasive margin of the cancer. Inter-observer differences were less than 5%, and the mean of the 3 values was obtained.

### Statistical analysis

Values for patient age, tumour size, and LIs for ERα, ERβ, PR, Ki-67 and $CD3^+$ cells were presented as mean ± 95% confidence interval (95% CI), and the association between immunoreactivity for EBAG9 and these parameters were evaluated using a Bonferroni test. Statistical differences between immunoreactivity for EBAG9 and menopausal status, stage, lymph node status, histological grade, mononuclear infiltration grade and HER-2/*neu* were evaluated in a cross-table using a $\chi^2$ test. Disease-free and overall survival analyses were calculated according to the Kaplan–Meier test. *P* values less than 0.05 were considered significant. The statistical significance of the differences in the survival analyses was calculated using the log-rank test.

### RESULTS

### Characterization of anti-EBAG9 antibody by Western blot analysis

The specificity of the anti-EBAG9 antibody was shown in Figure 1. Western blot analysis of human and mouse EBAG9 proteins revealed that the polyclonal antibody reacted with both in vitro translated human and mouse EBAG9 at 32 kDa. The band intensity of both human and mouse EBAG9 was abolished by pre-absorption according to the amount of added recombinant EBAG9 fusion protein in primary antibody before reaction with the membrane, suggesting that the reactivity of the antibody is specific to EBAG9 of both mouse and human.

### Immunohistochemistry

EBAG9 immunoreactivity was detected on the entire surface, and in the cytoplasm of invasive ductal carcinoma cells (Figure 2A).

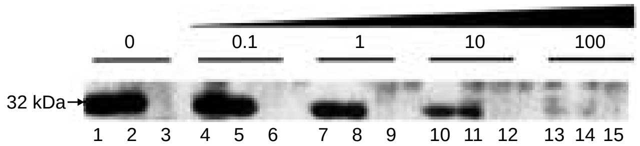

**Figure 1** Specificity of EBAG9 antibody by Western blot analysis. Lanes 3, 6, 9, 12 and 15 were loaded with a premix of TNT T7 Quick Coupled Transcription/Translation System as control. Lanes 1, 4, 7, 10 and 13 were loaded with in vitro translated mouse EBAG9 protein, and lanes 2, 5, 8, 11 and 14 were loaded with recombinant human EBAG9, as described in the Materials and Methods. Lanes 1 to 3 were incubated with 5 ml of 1:2000 diluted purified anti-EBAG9 antibody. Lanes 4 to 6, lanes 7 to 9, lanes 10 to 12, and lanes 13 to 15 were incubated with 5 ml of 1:2000 diluted purified anti-EBAG9 antibody, each of which was incubated before for 12 h at 4˚C with 0.1 µg, 1 µg, 10 µg, and 100 µg of GST-EBAG9 fusion protein, respectively. kDa; kilodalton

The number of cases immuno-positive for EBAG9 was 82 out of 91 cases (90.1%). In morphologically normal glands, immunoreactivity for EBAG9 was weakly detected on the luminal surface of epithelial cells (Figure 2B).

Immunoreactivity for ERα (Figure 2C), ERβ, and PR was detected in the nuclei of carcinoma cells, and that for Ki-67 was detected in the nuclei of carcinoma cells as well as some stromal cells. $CD3^+$ cells were distributed mainly in the invasive margin as well as in the stroma (Figure 3A, B), and the distribution pattern was similar to that of leukocytes by conventional H&E stain. In this study, HER-2/*neu* immunoreactivity was detected in the membranes of carcinoma cells in 39 out of 91 cases (42.9%).

### RT-PCR

As shown in Figure 4, mRNA expression for EBAG9, ERα and ERβ was detected as a specific single band (213 bp for EBAG9, 168 bp for ERα, and 228 bp for ERβ) in 7 out of 7 cases (100%), respectively. All the cases examined for RT-PCR analyses were immunohistochemically positive for EBAG9, ERα and ERβ.

### Relationship between EBAG9 and clinicopathological parameters

The results of association between EBAG9 and clinicopathological parameters are summarized in Table 1. As shown in Table 1, a significant inverse association was detected between EBAG9 immunoreactivity and mononuclear infiltration grade of the carcinoma tissue (*P* = 0.0020). There was, however, no significant relationship between EBAG9 immunoreactivity and patient age, menopausal status, stage, tumour size, lymph node status or histological stage.

### Relationship between EBAG9 and steroid receptors

Results of association between EBAG9 and immunohistochemical parameters are summarized in Table 2. There was a significant positive association between EBAG9 immunoreactivity and ERα LI (*P* = 0.0081). There was a significant inverse association between EBAG9 immunoreactivity and infiltration of $CD3^+$ cells (*P* = 0.0025). Other significant associations were not detected in this study.

### Relationship between primary and metastatic tumours

In the present investigation, lymph node metastasis was detected in 48 cases. As shown in Table 3, a significant association

© 2001 Cancer Research Campaign

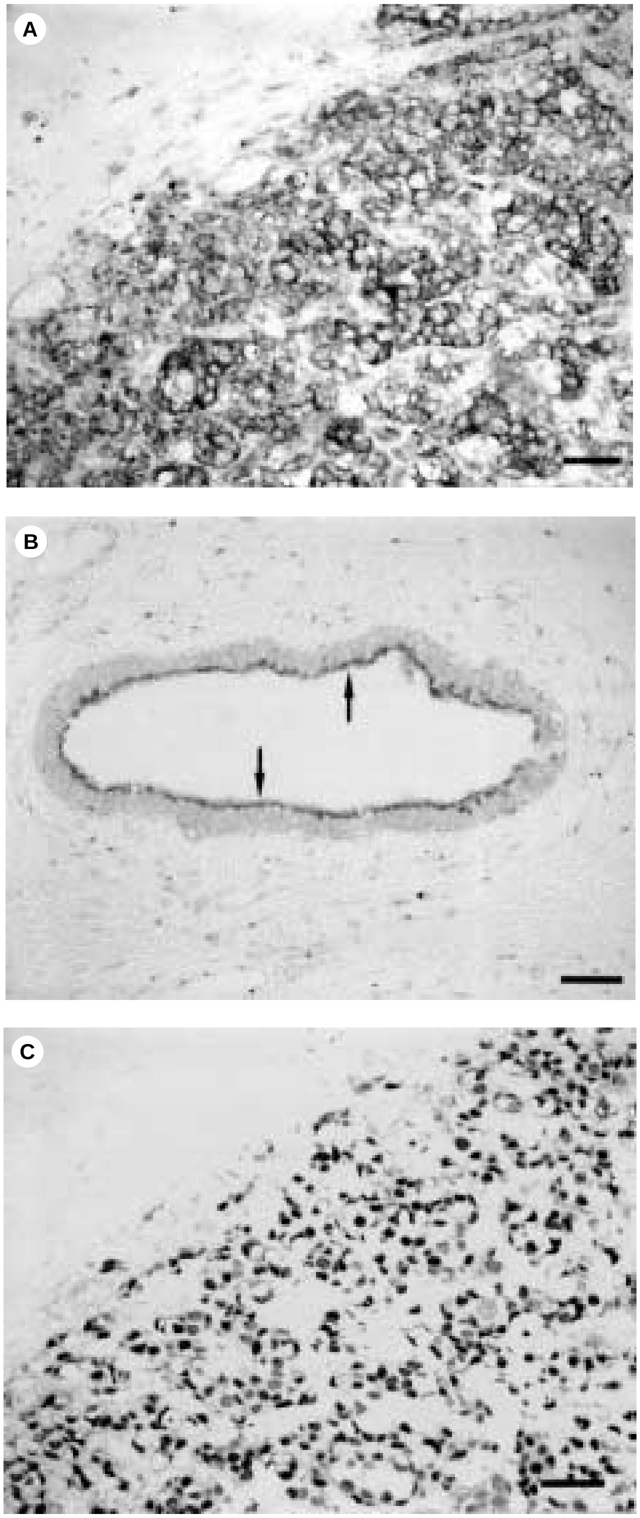

**Figure 2** Immunohistochemistry for EBAG9 in invasive ductal carcinoma. (**A**) EBAG9 immunoreactivity was detected on the entire surface and in the cytoplasm of invasive ductal carcinoma cells. (**B**) In morphologically normal mammary glands, immunoreactivity for EBAG9 was weakly detected on the luminal surface of epithelial cells (arrows). (**C**) Immunoreactivity for ER$\alpha$ was detected in the nuclei of carcinoma cells. The same field as (**A**). Original magnification $\times$ 140 and bar = 50 $\mu$m, respectively

($P$ = 0.031) was detected between immunoreactivity for EBAG9 in the primary lesion, and in the lymph node metastasis in these cases.

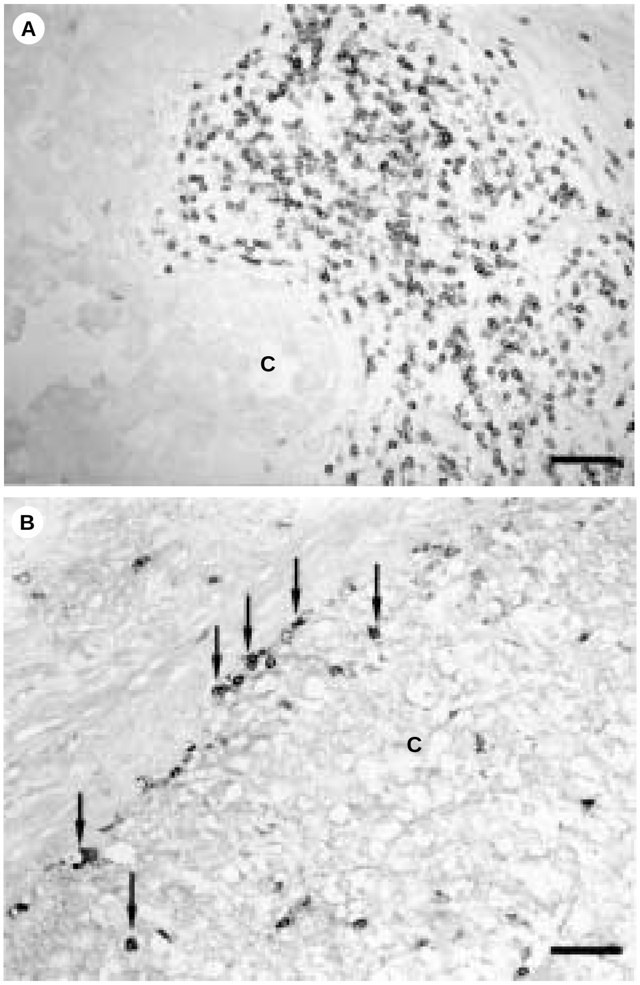

**Figure 3** Immunohistochemistry for CD3 in invasive ductal carcinoma. (**A**) Numerous CD3-positive cells were detected in the invasive margin of the breast carcinoma. In this case, immunoreactivity for EBAG9 was negative and ER$\alpha$ LI was 0. (**B**) Scattered CD3-positive cells were detected (arrows) in the invasive margin of the breast carcinoma. In this case, immunoreactivity for EBAG9 was positive, and ER$\alpha$ LI was 78 (the same case and same field as Figure 2A, C). (**C**) Carcinoma. Original magnification $\times$ 140 and bar = 50 $\mu$m, respectively

### Relationship between EBAG9 and clinical outcome

As shown in Table 4, no significant relationship was detected between EBAG9 immunoreactivity and disease-free ($P$ = 0.4882)

 *© 2001 Cancer Research Campaign*

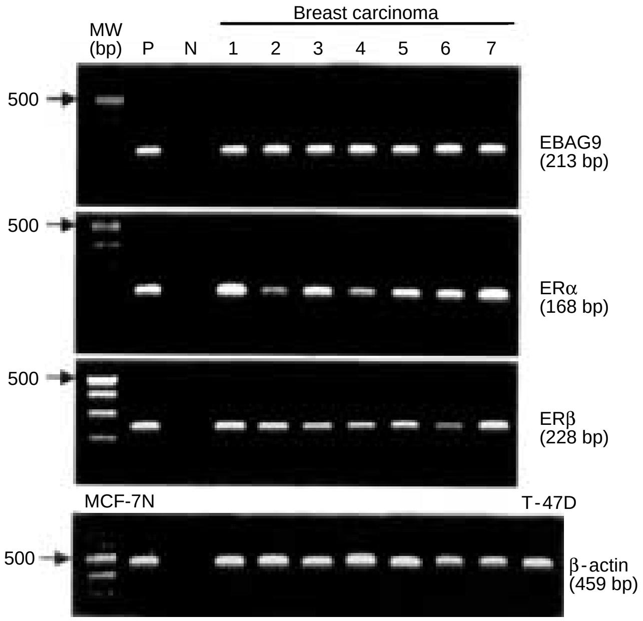

**Figure 4** Reverse transcription polymerase chain reaction (RT-PCR) analysis for EBAG9, ERα, and ERβ in 7 invasive ductal carcinomas. mRNA expression for EBAG9, ERα, ERβ, and β-actin was detected as a specific single band (213 bp for EBAG9, 168 bp for ERα, 228 bp for ERβ, and 459 bp for β-actin) in 7 out of 7 cases (100%), respectively. P, positive controls (MCF-7 cells for EBAG9 and ERα, and T-47D cells for ERβ); N, negative control (no cDNA substrate)

**Table 1** Association between EBAG9 immunoreactivity and clinicopathological parameters in human breast carcinomas

| | EBAG9 immunoreactivity | | *P* value |
|---|---|---|---|
| | + (*n* = 82) | – (*n* = 9) | |
| Age[a] (years) | 53.9 ± 1.4 | 51.6 ± 4.1 | NS |
| Menopausal status | | | |
| Premenopausal | 42 (46.2%) | 6 (6.6%) | |
| Postmenopausal | 40 (44.0%) | 3 (3.3%) | NS |
| Stage | | | |
| I | 16 (17.6%) | 3 (3.3%) | |
| II | 49 (53.8%) | 3 (3.3%) | |
| III | 12 (13.2%) | 2 (2.2%) | |
| IV | 5 ( 5.5%) | 1 (1.1%) | NS |
| Tumor size* (mm) | 28.7 ± 1.5 | 27.6 ± 3.7 | NS |
| Lymph node status | | | |
| Positive | 43 (47.3%) | 5 (5.5%) | |
| Negative | 39 (42.9%) | 4 (4.4%) | NS |
| Histological grade | | | |
| Grade I | 21 (23.1%) | 0 (0%) | |
| Grade II | 30 (33.0%) | 4 (4.4%) | |
| Grade III | 31 (34.1%) | 5 (5.5%) | NS |
| Mononuclear infiltration grade | | | |
| Grades 0 and 1 | 20 (22.0%) | 0 (0%) | |
| Grade 2 | 45 (49.5%) | 2 (2.2%) | |
| Grade 3 | 17 (18.7%) | 7 (7.7%) | 0.0020 |

[a]Data are presented as mean ± 95% confidence interval (95% CI). All other values represent the number of cases and their respective percentages. *P* values less than 0.05 were considered as significant.

or overall ($P$ = 0.6602) survival in the 91 invasive ductal carcinomas examined in this study.

**Table 2** Association between EBAG9 immunoreactivity and immunohistochemical parameters in human breast carcinomas

| | EBAG9 immunoreactivity | | *P* value |
|---|---|---|---|
| | + (*n* = 82) | – (*n* = 9) | |
| ER*α* LI[a] | 46.1 ± 3.9 | 13.3 ± 6.2 | 0.0081 |
| ER*β* LI[a] | 9.5 ± 1.2 | 2.6 ± 5.2 | NS |
| PR LI[a] | 43.0 ± 4.1 | 37.0 ± 14.4 | NS |
| Ki-67 LI[a] | 26.9 ± 1.7 | 25.3 ± 6.6 | NS |
| Infiltration of CD3⁺ cells[a] | 124.1 ± 6.5 | 189.5 ± 22.1 | 0.0025 |
| HER-2/*neu* | | | |
| Positive | 33 (36.3%) | 6 (6.5%) | |
| Negative | 49 (53.8%) | 3 (3.2%) | NS |

[a]Data are presented as mean ± 95% confidence interval (95% CI). All other values represent the number of cases and their respective percentages. *P* values less than 0.05 were considered as significant.

**Table 3** Association between EBAG9 immunoreactivity in the primary lesion and in lymph node metastasis of human breast carcinomas

| EBAG9 immunoreactivity in lymph node metastasis | EBAG9 immunoreactivity in primary lesion | | *P* value |
|---|---|---|---|
| | + (*n* = 43) | – (*n* = 5) | |
| + (*n* = 44) | 41 (85.5%) | 3 (6.3%) | |
| – (*n* = 4) | 2 ( 4.2%) | 2 (4.2%) | 0.031 |

Data represent the number of cases and their respective percentages. *P* value less than 0.05 was considered as significant.

**Table 4** Disease-free and overall survival analyses in breast cancer patients examined

| Variable | Disease-free survival *P* value | Overall survival *P* value |
|---|---|---|
| Lymph node status (positive/negative) | 0.0035* | < 0.0001* |
| Tumor size (> 20 mm/ ≦ 20 mm) | 0.0133* | 0.0115* |
| HER-2/*neu* (positive/negative) | 0.0647 | 0.0370* |
| Ki-67 LI (≧ 10/< 10) | 0.2099 | 0.1982 |
| Histological grade (III/I, II) | 0.2358 | 0.0376* |
| ERα LI (≧ 10/< 10) | 0.2500 | 0.3760 |
| EBAG9 immunoreactivity (+/–) | 0.4882 | 0.6602 |

Disease-free and overall survival analyses were calculated according to the Kaplan–Meier test. *P* values less than 0.05(*) were considered significant.

### DISCUSSION

In this study, EBAG9 immunoreactivity was detected in carcinoma cells in 82 out of 91 human breast carcinomas (90.1%). EBAG9 immunoreactivity was significantly associated with ERα LI ($P$ = 0.0081), and although association did not reach statistical significance, a similar tendency was also detected between EBAG9 and ERβ ($P$ = 0.074). ERβ has been demonstrated to activate gene transcription by binding to EREs of target genes (Enmark et al, 1997; Paige et al, 1999), as well as ERα. EBAG9 was isolated utilizing a genomic binding site cloning method from a cDNA library of MCF-7 human breast cancer cells (Watanabe et al, 1998), which expresses ERα and low level of ERβ (Vladusic et al, 2000). Transfection analyses have demonstrated that the nucleotide sequence between –86 to –36 contains an ERE

in the 5′-promoter region of the EBAG9 gene (Ikeda et al, 2000). mRNA levels of EBAG9 in MCF-7 cells are significantly increased within 6 hours of oestrogen treatment, an effect that is mediated by the binding of ERα to the ERE in the promoter of the EBAG9 gene (Ikeda et al, 2000). Results from our present study are consistent with these previous reports, and suggest that EBAG9 is widely distributed in carcinoma cells of human breast carcinoma tissues as a result of oestrogenic actions through ERα and/or ERβ.

It is well known that the expression of ERβ depends on the presence of ERα in the great majority of human breast cancers (Sasano et al, 1999; Skliris et al, 2001). However, the biological significance of ERβ remains undefined in the breast cancer. Previously, Speirs et al (1999) reported that breast tumours coexpressing ERα and ERβ tended to be node positive and higher grade using RT-PCR, and Dotzlaw et al (1999) have shown that ERβ mRNA level was lower in PR-positive breast cancer. However, immunohistochemical studies have demonstrated that ERβ-positive breast cancers were predominantly well-differentiated, node-negative and PR-positive (Jarvinen et al, 2000; Skliris et al, 2001). In our immunohistochemical study, ERβ LI was inversely correlated with histological grade ($P$ = 0.0030) or lymph node status ($P$ = 0.0059), and positively correlated with PR LI ($P$ = 0.0182). These present data are in good agreement with the latter reports. Considering that the former studies were conducted at the mRNA level, these different observations may partly reflect the discrepancy between mRNA and protein levels of ERβ.

There was a strong correlation between ERα LI and PR LI ($P$ < 0.0001) in this study, but no association was detected between EBAG9 immunoreactivity and PR LI ($P$ = 0.65). Transcription of the PR gene is enhanced and maintained by oestrogens, and a positive PR status has been regarded as one of the markers of functional oestrogenic pathways (Horwitz and Mcguire, 1978; Alexieva-Figusch et al, 1988). Therefore, these findings suggest a variety of oestrogenic actions, and different mechanisms may be involved in the regulation of expression of these 2 oestrogen-responsive genes in breast carcinomas.

EBAG9 immunoreactivity was detected on the entire surface and in the cytoplasm of invasive ductal carcinoma cells, while it was present only on the side of the luminal surface of normal glandular epithelia in this study. Similar findings have also been reported in some cancer-associated antigens, including carcinoembrionic antigen (CEA) and carbohydrate antigen 19-9 (CA19-9) (Hamada et al, 1985; Ichihara et al, 1988). These findings are in general regarded as ‘loss of polar distribution’ of membrane-associated antigens as a result of malignant transformation. Overproduction of CEA and CA19-9 in cancer cells may leak into the surrounding stroma and eventually result in an increment in their plasma levels (Hamada et al, 1985; Ichihara et al, 1988). EBAG9/RCAS1 has been identified as a membrane molecule which acts as a ligand in putative receptor present cells (Nakashima et al, 1999). Previously, Sonoda et al (1996) detected the EBAG9/RCAS1 protein in the vaginal discharges of EBAG9/RCAS1-positive cervical carcinoma patients. Therefore, EBAG9 protein may be secreted from breast carcinoma cells which in turn is likely to elevate the serum levels of EBAG9 in these patients. To date, no studies have examined the serum levels of EBAG9 protein in cancer patients, and the possible significance of EBAG9 as a serum tumour marker should be validated by further analyses in various human neoplasms including breast carcinoma.

In this study, EBAG9 immunoreactivity was inversely associated to infiltration of mononuclear cells ($P$ = 0.0020) and $CD3^+$ T lymphocytes ($P$ = 0.0025). Recently, Nakashima et al (1999) reported that activated $CD3^+$ T lymphocytes express a putative receptor for EBAG9/RCAS1, and that recombinant or soluble EBAG9/RCAS1 inhibits in vitro growth of these lymphocytes. In addition, several studies have demonstrated that the degree of intratumoural mononuclear cells and T lymphocytes are inversely correlated to ER-status (An et al, 1987; Whitford et al, 1990), but not to PR-status (An et al, 1987), in human breast carcinoma tissues. These findings were confirmed in the present study (inverse correlation between ERα LI and mononuclear infiltration grade ($P$ = 0.0012) or $CD3^+$ cell infiltration ($P$ = 0.0024), but no correlation between PR LI and mononuclear infiltration grade ($P$ = 0.29) or infiltration of $CD3^+$ cells ($P$ = 0.18)). An et al (1987) also reported that the number of other mononuclear subsets, including B lymphocytes, NK cells and macrophages, was relatively small and no significant correlations were detected with respect to ER or PR status. Results from the present study as well as those of previous investigations above suggest that EBAG9 secreted from carcinoma cells inhibits intratumoural T lymphocyte infiltration via oestrogenic actions in carcinoma cells through ER. Therefore, EBAG9 may play an important role in endocrine– immune interactions such as the immune escape of breast carcinoma cells.

It is well recognized that human cancer tissues are infiltrated with tumour-infiltrating lymphocytes (TIL) (Balch et al, 1990), a phenomenon known to be a manifestation of the host immune reaction to cancer cells (Rosenberg, 1996). TIL has been reported to be associated with improved prognosis of some carcinomas, including lung (DiPaola et al, 1977) and colon (Naito et al, 1998) carcinomas. However, the biological significance of TIL remains controversial in human breast carcinoma. For instance, prognosis of medullary-type breast carcinomas has been reported to be associated with the number of TIL (Moore and Foote, 1949). Black et al (1955) reported that 9 of 11 patients had high counts of TIL even when they had regional axillary lymph node metastases, and survived twice as long as patients without TIL. However, several groups have reported contradictory findings in that TIL was by no means correlated with clinical outcome of the patients (Champion et al, 1972) or histological grade of the tumour (An et al, 1987; Whitford et al, 1990). In this study, no significant correlations were detected between mononuclear infiltration grade or infiltration of $CD3^+$ cells and prognosis, or histological grade (data not shown), consistent with results of the later reports. In vitro studies of mononuclear cells isolated from breast carcinoma tissues have demonstrated the presence of suppressor activity capable of reducing immunologic responses (Vose et al, 1977; Vose and Moore, 1979). Further investigations are necessary to clarify whether inhibition of EBAG9 expression results in improved prognosis through the increased number of TIL in human breast carcinoma tissues.

In summary, we have demonstrated that EBAG9 is widely distributed in human breast carcinomas using immunohistochemistry and RT-PCR. Immunoreactivity for EBAG9 was significantly associated with ERα LI, and inversely associated with mononuclear infiltration grade or infiltration of $CD3^+$ T lymphocytes. Our present data suggest that EBAG9 is produced via ERs and inhibits the infiltration of T lymphocytes in human breast carcinomas in the possible spectrum of endocrine–immune interactions in patients with breast carcinoma.

### ACKNOWLEDGEMENTS

We appreciate the skilful technical assistance of Ms Kumiko Hidaka (Department of Pathology, Tohoku University School

 *© 2001 Cancer Research Campaign*

of Medicine). We would like to thank Mr Andrew Darnel (Department of Pathology, Tohoku University School of Medicine, Sendai, Japan) for the editing of this manuscript. This work was supported by a grant-in-aid for Cancer Research 7-1 from the Ministry of Health and Welfare, Japan, a grant-in-aid for scientific research area on priority area (A-11137301) from the Ministry of Education, Science and Culture, Japan, a grant-in-aid for Scientific Research (B-11470047) from the Japan Society for the Promotion of Science, and a grant from The Naitou Foundation and Suzuken Memorial Foundation.

### REFERENCES

Alexieva-Figusch J, Van Putten WL, Blankenstein MA, Blonk-Van Der Wijst J and Klijn JG (1988) The prognostic value and relationships of patient characteristics, estrogen and progestin receptors, and site of relapse in primary breast cancer. *Cancer* **61**: 758–768

An T, Sood U, Pietruk T, Cummings G, Hashimoto K and Crissman JD (1987) In situ quantitation of inflammatory mononuclear cells in ductal infiltrating breast carcinoma. Relation to prognostic parameters. *Am J Pathol* **128**: 52–60

Balch CM, Riley LB, Bae YJ, Salmeron MA, Platsoucas CD, von Eschenbach A and Itoh K (1990) Patterns of human tumor-infiltrating lymphocytes in 120 human cancers. *Arch Surg* **125**: 200–205

Black MM, Opler SR and Speer FD (1955) Survival in breast cancer cases in relation to the structure of the primary tumor and regional lymph nodes. *Surg Gynecol Obstet* **98**: 543–551

Bloom HJG and Richardson WW (1957) Histological grading and prognosis in breast cancer. A study of 1409 cases of which 359 have been followed for 15 years. *Br J Cancer* **11**: 359–377

Champion HR, Wallace IW and Prescott RJ (1972) Histology in breast cancer prognosis. *Br J Cancer* **26**: 129–138

DiPaola M, Bertolotti A, Colizza S and Coli M (1977) Histology of bronchial carcinoma and regional lymph nodes as putative immune response of the host to the tumor. *J Thorac Cardiovasc Surg* **73**: 531–537

Dotzlaw H, Leygue E, Watson PH and Murphy LC (1999) Estrogen receptor-beta messenger RNA expression in human breast tumor biopsies: relationship to steroid receptor status and regulation by progestins. *Cancer Res* **59**: 529–532

Elston CW and Ellis IO (1991) Pathological prognostic factors in breast cancer. I. The value of histological grade in breast cancer. Experience from a large study with long-term follow-up. *Histopathology* **19**: 403–410

Enmark E, Pelto-Huikko M, Grandien K, Lagercrantz S, Lagercrantz J, Fried G, Nordenskjold M and Gustafsson JA (1997) Human estrogen receptor beta-gene structure, chromosomal localization, and expression pattern. *J Clin Endocrinol Metab* **82**: 4258–4265

Hamada Y, Yamamura M, Hioki K, Yamamoto M, Nagura H and Watanabe K (1985) Immunohistochemical study of carcinoembryonic antigen in patients with colorectal cancer. Correlation with plasma carcinoembryonic antigen levels. *Cancer* **55**: 136–141

Hamlin IM (1968) Possible host resistance in carcinoma of the breast: a histological study. *Br J Cancer* **22**: 383–401

Horwitz KB and McGuire WL (1978) Estrogen control of progesterone receptor in human breast cancer. Correlation with nuclear processing of estrogen receptor. *J Biol Chem* **253**: 2223–2228

Ichihara T, Nagura H, Nakao A, Sakamoto J, Watanabe T and Takagi H (1988) Immunohistochemical localization of CA 19–9 and CEA in pancreatic carcinoma and associated diseases. *Cancer* **61**: 324–333

Ikeda K, Sato M, Tsutsumi O, Tsuchiya F, Tsuneizumi M, Emi M, Imoto I, Inazawa J, Muramatsu M and Inoue S (2000) Promoter analysis and chromosomal mapping of human EBAG9 gene. *Biochem Biophys Res Commun* **273**: 654–660

Iwasaki T, Nakashima M, Watanabe T, Yamamoto S, Inoue Y, Yamanaka H, Matsumura A, Iuchi K, Mori T and Okada M (2000) Expression and prognostic significance in lung cancer of human tumor-associated antigen RCAS1. *Int J Cancer* **89**: 488–493

Jarvinen TA, Pelto-Huikko M, Holli K and Isola J (2000) Estrogen receptor beta is coexpressed with ERalpha and PR and associated with nodal status, grade, and proliferation rate in breast cancer. *A J Pathol* **156**: 29–35

Kaku T, Sonoda K, Kamura T, Hirakawa T, Sakai K, Amada S, Ogawa S, Kobayashi H, Nakashima M, Watanabe T and Nakano H (1999) The prognostic significance of tumor-associated antigen 22–1–1 expression in adenocarcinoma of the uterine cervix. *Clin Cancer Res* **5**: 1449–1453

Kuiper GG, Enmark E, Pelto-Huikko M, Nilsson S and Gustafsson JA (1996) Cloning of a novel receptor expressed in rat prostate and ovary. *Proc Nat Acad Sci USA* **93**: 5925–5930

Moore OS Jr. and Foote FW Jr., (1949) The relatively favorable prognosis of medullary carcinoma of the breast. *Cancer* **2**: 635–642

Mosselman S, Polman J and Dijkema R (1996) ER beta: identification and characterization of a novel human estrogen receptor. *FEBS Letters* **392**: 49–53

Naito Y, Saito K, Shiiba K, Ohuchi A, Saigenji K, Nagura H and Ohtani H (1998) $CD8^+$ T cells infiltrated within cancer cell nests as a prognostic factor in human colorectal cancer. *Cancer Res* **58**: 3491–3494

Nakashima M, Sonoda K and Watanabe T (1999) Inhibition of cell growth and induction of apoptotic cell death by the human tumor-associated antigen RCAS1. *Nature Med* **5**: 938–942

Ogawa S, Inoue S, Watanabe T, Hiroi H, Orimo A, Hosoi T, Ouchi Y and Muramatsu M (1998a) The complete primary structure of human estrogen receptor beta (hER beta) and its heterodimerization with ER alpha in vivo and in vitro. *Biochem Biophys Res Commun* **243**: 122–126

Ogawa S, Inoue S, Watanabe T, Orimo A, Hosoi T, Ouchi Y and Muramatsu M (1998b) Molecular cloning and characterization of human estrogen receptor betacx: a potential inhibitor of estrogen action in human. *Nucl Acid Res* **26**: 3505–3512

Paige LA, Christensen DJ, Gron H, Norris JD, Gottlin EB, Padilla KM, Chang CY, Ballas LM, Hamilton PT, McDonnell DP and Fowlkes DM (1999) Estrogen receptor (ER) modulators each induce distinct conformational changes in ER alpha and ER beta. *Proc Natl Acad Sci USA* **96**: 3999–4004

Ponte P, Ng SY, Engel J, Gunning P and Kedes L (1984) Evolutionary conservation in the untranslated regions of actin. *Nucleic Acids Res* **12**: 1687–1696

Pujol P, Rey JM, Nirde P, Roger P, Gastaldi M, Laffargue F, Rochefort H and Maudelonde T (1998) Differential expression of estrogen receptor-alpha and -beta messenger RNAs as a potential marker of ovarian carcinogenesis. *Cancer Research* **58**: 5367–5373

Rosenberg SA (1996) The immunotherapy of solid cancers based on cloning the genes encoding tumor-rejection antigens. *Ann Rev Med* **47**: 481–491

Sasano H, Suzuki T, Matsuzaki Y, Fukaya T, Endoh M, Nagura H and Kimura M (1999) Messenger ribonucleic acid in situ hybridization analysis of estrogen receptors alpha and beta in human breast carcinoma. *J Clin Endocrinol Metab* **84**: 781–785

Skliris GP, Carder PJ, Lansdown MR and Speirs V (2001) Immunohistochemical detection of ERbeta in breast cancer: towards more detailed receptor profiling? *Br J Cancer* **84**: 1095–1098

Sonoda K, Nakashima M, Kaku T, Kamura T, Nakano H and Watanabe T (1996) A novel tumor-associated antigen expressed in human uterine and ovarian carcinomas. *Cancer* **77**: 1501–1509

Speirs V, Parkes AT, Kerin MJ, Walton DS, Carleton PJ, Fox JN and Atkin SL (1999) Coexpression of estrogen receptor alpha and beta: poor prognostic factors in human breast cancer? *Cancer Res* **59**: 525–528

Takeyama J, Suzuki T, Inoue S, Kaneko C, Nagura H, Harada N and Sasano H (2001) Expression and cellular localization of estrogen receptors alpha and beta in human fetus. *J Clin Endocrinol Metab* **86**: 2258–2262

Thomas DB (1984) Do hormones cause cancer? *Cancer* **53**: 595–604

Tsai MJ and O'Malley BW (1994) Molecular mechanisms of action of steroid/thyroid receptor superfamily members. *Ann Rev Biochem* **63**: 451–486

Tsuchiya F, Ikeda K, Tsutsumi O, Hiroi H, Momoeda M, Taketani Y, Muramatsu M and Inoue S (2001) Molecular cloning and characterization of mouse EBAG9, homolog of a human cancer associated surface antigen: expression and regulation by estrogen. *Biochem Biophys Res Commun* in press

Vihko R and Apter D (1989) Endogenous steroids in the pathophysiology of breast cancer. *CRC Crit Rev Oncol/Hematol* **9**: 1–16

Vladusic EA, Hornby AE, Guerra-Vladusic FK, Lakins J and Lupu R (2000) Expression and regulation of estrogen receptor beta in human breast tumors and cell lines. *Oncol Rep* **7**: 157–167

Vose BM and Moore M (1979) Suppressor cell activity of lymphocytes infiltrating human lung and breast tumours. *Int J Cancer* **24**: 579–585

Vose BM, Vanky F and Klein E (1977) Human tumour—lymphocyte interaction in vitro. V. Comparison of the reactivity of tumour-infiltrating, blood and lymph-node lymphocytes with autologous tumour cells. *Int J Cancer* **20**: 895–902

Watanabe T, Inoue S, Hiroi H, Orimo A, Kawashima H and Muramatsu M (1998) Isolation of estrogen-responsive genes with a CpG island library. *Mol Cell Biol* **18**: 442–449

Whitford P, Mallon EA, George WD and Campbell AM (1990) Flow cytometric analysis of tumour infiltrating lymphocytes in breast cancer. *Br J Cancer* **62**: 971–975

© 2001 Cancer Research Campaign
